# 

**Published:** 1876-10

**Authors:** 


### BOOKS AND PAMPHLETS RECEIVED.

### BOOKS.

Chirurgie-Antiseptique, principes, modes d'application et résultats du pansement de Lister. By Le dr. J. Lucas-Championnière.

A Manual of Percussion and Auscultation; of the physical diagnoses of the lungs and heart, and of thoracic aneurism. By Austin Flint, M.D., etc.

A Manual of Midwifery. Second American, from third London edition; revised and enlarged. By Alfred Meadows, M.D., etc.

The Theory and Practice of Medicine. Second American edition. By F. T. Roberts, M.D., etc.

On Tracheotomy, especially in relation to diseases of the Larynx and Trachea. By W. Pugin Thornton, Surgeon, etc.

Diseases of the Bladder, Prostate Gland and Urethra, etc. Being the fourth edition of the "Irritable Bladder," revised and enlarged. By Frederick James Gant, F.R.C.S., etc.

Hay Fever, or Summer Catarrh, etc. By Geo. M. Beard, A.M., M.D., etc.

Studies, chiefly clinical, on the non-emetic use of Ipecacuanha, etc. By A. A. Woodhull, M.D., etc.

Physicians' Visiting List and Complete Pocket Record. Prepared by James I. Hale, M.D., Anna, Ill. 1876.

Transactions of the College of Physicians of Philadelphia. Third Series. Vol. II. 1876.

A Treatise on the Science and Practice of Midwifery. By W. S. Playfair, M.D., F.R.C.P., etc.

PAMPHLETS.

Report on Vaccination, being an inquiry concerning Human Vaccine, Vaccino-Syphilis and Animal Vaccine, etc. By Wm. B. Davis, A.M., M.D., etc.

A Clinical Lecture on the use of Plastic Dressing in fractures of the lower extremities. By D. W. Yandell, M.D., etc.

Transactions of the Medical Association of the State of Missouri. 1876.

An Address on some of the leading Public Health Questions; with remarks on the extent of swamp lands in the United States and their reclamation as a sanitary and economic measure. By J. M. Toner, M.D., etc.

Annual Announcements for 1876 of the National Medical College of the Columbian University, Washington, and Hahnemann Medical College and Hospital, Chicago, Ill.

Report of the Committee on Medical Education, made to the Medical Society of the State of California. By J. F. Montgomery, M.D., Chairman, etc. 1876.

Transactions of the Medical Association of the State of Alabama. 1876.

Western North Carolina as a health resort. By W. Gleitsmann, M.D. 1876.

The Climatotherapy of the American Mountain Sanitarium for Consumption. By S. E. Chaillé, A.M., M.D., etc. 1876.

Third Annual Announcement of the College of Physicians and Surgeons of Indiana, Indianapolis. 1876–7.

### ANNOUNCEMENTS FOR THE MONTH.

**MONDAYS.** Societies.

*Mondays, Oct.* 2 *and* 16.—Chicago Medical Society. Regular meetings.
*Mondays, Oct.* 9 *and* 23.—Chicago Soc. of Physicians and Surgeons. Regular meetings.

Clinics. *Every Monday.*

At Eye and Ear Infirmary, 2 P. M.—Prof. Holmes.
At Central Dispensary (Wood and Harrison sts.), 2 P.M. *Gynecological*—Dr. Adolphus; 3, *Medical*—Dr. Bridge; 3, *Dermatological*—Dr. W. J. Maynard.
At Mercy Hospital, 2 P. M. *Medical*—Prof. Johnson.
At Chicago College, 2 P. M. *Gynecological*—Prof. Merriman.

Lectures. *Every Monday.*

At Rush Medical College (Harrison and Wood sts.)—9½ to 12½ o'clock, Profs. Freer, Lyman and Powell; 4 to 6, Profs. Gunn and Haines. At Chicago College—8½ to 12½, Profs. Jewell, Hyde, Hatfield and Bond; 3 to 6, Profs. Nelson and Davis, Quine and Andrews, and Roler.

**TUESDAYS.** Societies.

*Tuesday, Oct,* 10.—Academy of Science. Regular meeting at 8 P.,M. (263 Wabash av.)
*Tuesday, Oct.* 26.—Medico-Historical Society. Regular meeting, 8 P. M.

Clinics. *Every Tuesday.*

At County Hospital, 2 P. M.; *Medical*—Prof. Ross. 3 P. M.; *Surgical*—Prof. Powell.
At Mercy Hospital, 2 P. M.; *Medical*—Prof. Hollister.
At Chicago College, 2 P. M,; *Gynecological*—Prof. Roler.
At Central Dispensary, 2, *Surgical*—Dr. Graham. 2, *Diseases of Chest*—Dr. Ingals.

Lectures. *Every Tuesday.*

At Rush College—9½ to 12½, Profs. Miller, Allen and Parkes; 4 to 6, Profs. Gunn and Haines. At Chicago College—8½ to 12½, Profs. Jewell, Quine, Merriman and Bond; 3 to 6, Profs. Davis and Nelson, Andrews and Hatfield, and Byford.

**WEDNESDAYS.** Clinics. *Every Wednesday.*

At County Hospital—2 P. M., *Gynecological*, Prof. Fitch; 2 P. M., *Ophthalmological*, Dr. Montgomery.
At Chicago College—2 P. M., *Gynecological*, Prof. Nelson.
At Mercy Hospital—2 P. M., *Surgical*, Prof. Andrews.
At Central Dispensary—2 P. M., *Gynecological*, Prof. Etheridge; 3 P. M., *Medical*, Dr. Bridge; 3 P. M., *Dermatological*, Dr. Maynard.

Lectures. *Every Wednesday.*

At Rush College—9½ to 12½, Profs. Freer, Allen and Parkes; 4 to 6, Profs. Etheridge and Haines. At Chicago College—8½ to 12½, Profs. Hyde, Isham, Hatfield and Bond; 3 to 6, Profs. Davis and Curtis, Quine and Jones, and Roler.

**THURSDAYS.** Clinics. *Every Thursday.*

At Mercy Hospital—2 P. M., *Medical*, Prof. Johnson.
At Rush College—2 P. M., *Medical*, Prof. Ross; 3 P. M., *Diseases of the Nervous System*, Prof. Lyman.
At Chicago College—2 P. M., *Gynecological*, Prof. Merriman.
At Central Dispensary—2 P.M., *Surgical*, Dr. Graham; 2, *Diseases of Chest*, Dr. Ingals.

Lectures. *Every Thursday.*

At Rush College—9½ to 12½, Profs. Miller, Allen and Parkes; 4 to 6, Profs. Gunn and Etheridge. At Chicago College—8½ to 12½, Profs. Hollister, Isham, Merriman and Bond; 3 to 6, Profs. Davis and Nelson, Andrews and Hatfield, and Byford.

**FRIDAYS.** Societies.

*Friday, Oct.* 13.—State Microscopical Society of Illinois. Regular meeting, 8 P. M.

Clinics. *Every Friday.*

At County Hospital—2 P. M., *Medical*, Prof. Ross; 3 P. M., *Surgical*, Prof. Powell.
At Mercy Hospital—2 P. M., *Ophthalmological*, Prof. Jones.
At Chicago College—2 P. M., *Gynecological*, Prof. Roler.
At Central Dispensary—2 P. M., *Gynecological*, Dr. Adolphus.

Lectures. *Every Friday.*

At Rush College | 9½ to 12½, Profs. Freer, Allen and Parkes; 4 to 6, Profs. Gunn and Holmes. At Chicago College—8½ to 12½, Profs. Hollister, Isham, Hatfield and Bond 3 to 6. Profs. Davis and Nelson, Jones and Quine, and Roler.

**SATURDAYS.** Clinics. *Every Saturday.*

At Chicago College—2 P. M., *Surgical*, Prof. Andrews or Isham; *Gynecological*, Prof. Nelson; 3 P. M., *Medical*, Prof. Davis.
At Rush College—2 P. M., *Surgical*, Prof. Gunn.

Lectures. *Every Saturday.*

At Rush College—9½ to 12½, Profs. Miller, Lyman and Etheridge. At Chicago College 8½ to 11½, Profs. Hollister, Quine and Hyde; 3 to 6, Profs. Curtis, Andrews and Quine and Byford.

At all the above named Clinics visiting regular practitioners are, we believe, admitted.

At the South Side Dispensary (Chicago College) there are six daily special Clinics, for sections of the classes of the Chicago College.

[The schedule of the Woman's Medical College had not been received on going to press.—Ed.]

